# Mental health of undocumented migrants and migrants undergoing regularization in Switzerland: a cross-sectional study

**DOI:** 10.1186/s12888-021-03149-7

**Published:** 2021-04-01

**Authors:** Julien Fakhoury, Claudine Burton-Jeangros, Liala Consoli, Aline Duvoisin, Delphine Courvoisier, Yves Jackson

**Affiliations:** 1 Swiss NCCR “LIVES - Overcoming Vulnerability: Life Course Perspectives”, University of Geneva, Geneva, Switzerland; 2 Center for the Interdisciplinary Study of Gerontology and Vulnerability, University of Geneva, Geneva, Switzerland; 3 Institute of sociological research, University of Geneva, Geneva, Switzerland; 4 Division of Rheumatology, University Hospital of Geneva, Geneva, Switzerland; 5 Division of Primary Care Medicine, Geneva University Hospital and University of Geneva, Rue Gabrielle Perret Gentil 4, 1211, 14 Geneva, Switzerland

**Keywords:** Mental health, Anxiety, Depression, Undocumented migrants, Regularization, Policy

## Abstract

**Background:**

Undocumented migrants live and work in precarious conditions. Few studies have explored the mental health consequences of such environment. The objective of this study is to describe the mental health of migrants at different stages of a regularization program.

**Methods:**

This cross-sectional study included migrants undocumented or in the process of regularization. We screened for symptoms of anxiety, depression and sleep disturbance using validated tools. We created a composite outcome of altered mental health including these components plus self-report of a recent diagnosis of mental health condition by a health professional.

**Results:**

We enrolled 456 participants of whom 246 (53.9%) were undocumented. They were predominantly women (71.9%) with a median age of 43.3 (interquartile range: 15.5) years, from Latin America (63.6%) or Asia (20.2%) who had lived in Switzerland for 12 (IQR: 7) years. Overall, 57.2% presented symptoms of altered mental health. Prevalence of symptoms of anxiety, depression and sleep disturbance were 36% (95% confidence interval: 31.6–40.6%), 45.4% (95% CI: 40.8–50.1%) and 23% (95% CI: 19.2–27.2), respectively. Younger age (adjusted odd ratio: 0.7; 95% CI: 0.5–0.9 for each additional decade), social isolation (aOR: 2.4; 95% CI: 1.4–4.2), exposure to abuse (aOR: 1.9; 95% CI: 1.1–3.5), financial instability (aOR: 2.2; 95% CI: 1.4–3.7) and multi-morbidity (aOR: 3.2; 95% CI: 1.7–6.5) were associated with increased risk of having altered mental health while being in the early stages of the process of regularization had no effect (aOR: 1.3: 95% CI: 0.8–2.2).

**Conclusions:**

This study highlights the need for multi-pronged social and health interventions addressing the various domains of undocumented migrants living difficulties as complement to legal status regularization policies. Protection against unfair working conditions and abuse, access to adequate housing, promoting social integration and preventive interventions to tackle the early occurrence of chronic diseases may all contribute to reduce the burden of altered mental health in this group. More research is needed to assess the long-term impact of legal status regularization on mental health.

## Background

International migration steadily increases in the context of economic, political and environmental instability. In 2019, it concerned 272 million people, which represented 3.5% of the World’s population [1]. Undocumented migrants live in a country of destination without valid residency permit. Pathways to this situation include overstaying an entry visa, entering a country without valid travel documentation, losing valid residency permit or being born of undocumented parents [1]. Most are international migrant workers and failed asylum seekers account for a lesser stock. The population of such migrant is not precisely known but estimates pointed to 6 million people in Europe [2]. While a body of laws and conventions define the rights of regular migrants, refugees and asylum seekers, undocumented migrants remain mostly unprotected. Their limited access to civil and social rights, essential services and resources exposes them to diverse post-migration difficulties [1, 3]. They are frequently employed in informal, low-skill, precarious and hazardous jobs and are susceptible to work-related abuse and exploitation [4]. Access to medical services varies between countries and is frequently limited to emergency situations [5]. Overall, these factors tend to negatively impact on health and wellbeing of undocumented migrants [5–7].

In Europe, there is only limited evidence about undocumented migrants’ mental health and its determinants [5]. Most studies have reported data from small samples of participants, frequently retrospectively, and often collected in healthcare setting failing to reflect the situation in the community [6]. Indeed, several factors limit research in this hard-to-reach population such as gaining trust and participation, adequately sampling and being able to comprehensively assess mental health taking account the unique social and living conditions undocumented migrants face [5, 8, 9]. While they appear to suffer less severe and frequent mental health conditions than asylum seekers and refugees, their mental health status is not as good as in the general population [10, 11]. Anxiety and depression have been reported as the most prevalent mental health problems faced by undocumented migrants in Europe while post-traumatic stress disorder (PTSD) affects more frequently migrants with a former asylum seeker or refugee background [6, 10, 12, 13]. Studies conducted among asylum seekers and refugees have highlighted how post-migration mental health stressors evolved with the duration of residency in the country of destination. Indeed, while socioeconomic factors predominated in the early settlement period, loneliness and problems with adjustment to life in the country of destination were most prominent factors later on [14]. The few studies conducted among undocumented migrants in Europe have only reported about a limited range of such factors. In Spain, migrant reported heavy workload, insufficient income and the lack of formal working contract due to their undocumented position as major preoccupations for their health [15, 16]. In Italy and Sweden, male gender, older age, precarious socioeconomic conditions, including housing insecurity, feeling physically insecure and the presence of medical comorbidities along with difficulties accessing to healthcare put migrants at higher risks of suffering a mental health condition [12, 17].^14-15^A qualitative study conducted in Norway showed that while higher educational attainment was associated with less psychological distress, migrants having more people financially at charge and those exposed to abuse and discrimination showed worse mental health status [18]. To date, no study has reported about differences in mental health status and related factors among undocumented migrants at different stage of their integration process in Europe. While better integration into the host society through the reception of a residency permit has been associated with better mental health status in refugees in Western countries and in youth in the United States, no study has so far explored this topic in undocumented adults Europe [19, 20]. The burden of mental health conditions and the factors associated may differ at different stage of the integration process and thus require distinct social and health policies. The study hypothesis is that undocumented migrants at a more advanced stage of integration as attested by the eligibility to receive a residency permit may have lower prevalence of mental health conditions. So far, there is a dearth of evidence about the impact of programs aiming to promote mental health in undocumented and recently documented migrants. This paper aims to contribute to bridge these gaps and to allow policy-makers and public health planners to better tackle this major health problem. .

The Parchemins Study aims to prospectively monitor the health impact of a legal status regularization policy for undocumented migrants in Geneva, Switzerland [21]. The objective of this paper is to report about undocumented migrants’ mental health and its determinants during the first study wave.

## Methods

### Setting

Switzerland hosts 50–100′000 undocumented migrants of whom 10–15′000 live in the Canton of Geneva (population: 500′000) [22]. They are predominantly immigrant workers without valid residency permit while failed asylum-seekers represent a tiny minority [23]. Middle-aged women working in domestic work predominate and most originate from Latin America and the Philippines, while those coming from the Balkan region, North or West Africa are less numerous. These migrants are entitled to purchase a private health insurance to access to the healthcare system, but less than 10% are effectively insured due to administrative and financial barriers [23]. Their main port of entry within the healthcare system is a dedicated unit of the Geneva University Hospital (HUG). In 2017, the Geneva Canton Council implemented a pilot policy (Operation Papyrus) to provide residency permit (regularization) to undocumented migrants upon stringent conditions. This two-year program targeted non-European Union and non-European Free Trade Association undocumented migrants who met the following criteria: a) a continuous stay in Geneva for 10 years for individuals or 5 years for families with school-aged children; b) basic French oral proficiency; c) sufficient financial resources; d) lack of criminal record other than the absence of residency permit; and e) absence of previous application to seek asylum in Switzerland [24]. Eligible undocumented migrants applied for the permit with the support of workers’ unions and non-governmental organizations (NGO) mandated by the Canton Council to act as gatekeepers into the regularization process. This process was designed to ensure that migrants who fulfilled all the eligibility criteria at the time of application would be granted a residency permit.

### Study design

This cross-sectional survey within the prospective Parchemins study reports the results of the first wave of data collection which took place in 2018. Prospective data collection will unfold over four annual waves (2018–2021). The study sample includes participants with two distinct administrative situations: a) previously undocumented migrants who had received a residency permit within 3 months prior to inclusion and those meeting all eligibility criteria whose application for regularization is still being processed; and b) eligible undocumented migrants unwilling to regularize their situation or migrants who are lacking one or more criteria to apply for regularization. The full protocol of the Parchemins study can be consulted elsewhere [21].

We developed a questionnaire which included validated scales and measurements and new items. We pre-tested it on a sub-sample of participants using different languages. Field investigators were fluent in the different languages used in data collection and trained and supervised by a senior staff during their first interviews.

### Recruitment

In order to set up a diverse sample of undocumented migrants living in Geneva, we used various information and recruitment strategies in different settings. First, undocumented migrants were informed about the study during information meetings about Operation Papyrus in the community; second, we made direct contacts within the Operation Papyrus partner organizations offices during free legal consultation clinics; third, we provided information in the waiting room of the dedicated healthcare facility at HUG; fourth, information was advertised through diverse social media, including a Facebook page; fifth, newly recruited participants were asked to inform their peers about the study (snowball) [21].

### Inclusion criteria

Migrants aged ≥18 years, originating from countries outside the European Union or the European Free Trade Association, who had been living in Geneva without a valid residency permit (undocumented) for at least 3 years or who had received a residency permit through Operation Papyrus in the previous three months and who had not applied for asylum in Switzerland or were not former legal refugees were eligible to participate. In addition, we included only those planning to remain in Geneva for three more years not only for follow-up reason but also as a marker of social and financial stability. The three months cut-off period after regularization was determined by consensus with key community informants. We hypothesized that this timeframe was too short to bring significant changes in the living (accommodation), working (type of jobs and income), and social condition of newly regularized migrants susceptible to significantly modify the main outcomes.

### Participants

Participants were interviewed at the location of their choice in Geneva, Switzerland. The questionnaire was administered face-to-face in French, Spanish, Portuguese, or English, which corresponds to the main languages spoken by undocumented migrants in Geneva [22].

### Variables

The primary outcome was altered mental health. We defined it as a composite outcome to reflect the complex situation in which undocumented migrants live. It was defined as the presence of either positive screening for symptoms of anxiety, depression, or sleep disturbance, or having been diagnosed recently with anxiety or depression by a health professional. It was intended to provide a global overview of the burden of altered mental health in this population. The secondary outcomes were prevalence of positive screening for symptoms of depression, anxiety, sleep disturbance or recent diagnosis of a mental health condition by a health professional.

We selected screening tools for mental health based on their validity and reliability in clinical and non-clinical settings. The 9-item patient health questionnaire (PHQ-9) is a self-administered questionnaire that explores the occurrence of common depressive symptoms in the last 14 days [25]. Participants grade the symptoms on a 4-point scale from never (0 point) to almost every day (3 points). The total score ranges from 0 to 27 points and a score equal or above 5 indicates the presence of a major depressive disorder. It can be used to grade the severity of the depressive disorder using cut-off scores of 5, 10, 15 and 20 representing mild, moderate, moderately severe and severe levels of depressive symptoms. This questionnaire has a good accuracy (sensitivity and specificity of 88%) and internal consistency (alpha-Cronbach of 0.86–0.89 and 0.81in our sample) and has been used in large population-based cohorts in non-clinical setting [25].

The 7-item general anxiety disorder questionnaire (GAD-7) explores common anxiety symptoms’ occurrence in the last 14 days that participants grade the same way as the PHQ-9 for a total score ranging from 0 to 21 points. Five or more points indicate the presence of symptoms of anxiety and cut-offs of 5, 10 and 15 represent mild, moderate and severe levels of these symptoms. Sensitivity and specificity for the diagnosis of general anxiety disorder are 89 and 82%, respectively with an internal consistency of 0.92 (0.84 in our sample) [26, 27]. It has been used in various studies conducted among the general population. Screening for sleep disturbance relied on the sixth item of the Pittsburgh Sleep Quality Index (PSQI) which asks participants to grade their sleep quality over the past month on a four-level scale [28]. Participants choosing fairly bad or bad were considered as presenting sleep disturbance [29]. The PSQI is one of most frequently used sleep quality evaluation tool. It shows a strong validity and reliability to screen for sleep dysfunction in clinical and non-clinical samples with an internal consistency of 70–83% [29].

Independent variables were selected on the basis of findings from previous studies in this field. They encompassed migration, economic, social, and health dimensions. Migration-related variables included the current legal status regarding residency in Switzerland (regularized or in process of regularization vs. lack of residence permit), duration of stay in Geneva (continuous variable) and the maintenance of active connection with the country of origin as assessed by at least one visit to home country since arriving in Switzerland (No vs. Yes). Financial reserve was assessed as the ability to pay a CHF 1500 (Euro 1450, $ 1500) bill at short notice (Able vs. Unable). This question is used in population surveys in Switzerland using a higher threshold (CHF 2500) as the median income of undocumented migrants is well below this of the general Swiss population [30]. We calculated a housing crowding index on the basis of the number of occupants per bedrooms in each accommodation. A ratio > 2 indicated overcrowding [31]. Social integration was assessed by exploring the size of the social network (ie. the number of significant persons participants could rely on in case of distress) and self-assessment of social isolation (Rather or very connected vs. Rather or very isolated). Using two distinct dichotomous variables, we also explored exposure in the last 12 months to discrimination related to the nationality or ethnic origin or to verbal, physical or sexual abuse (No vs. Yes). Regarding health, participants were asked about their access to the healthcare system through the purchase of a private health insurance and the presence of common chronic medical conditions in accordance with the Swiss health survey [32]. Multi-morbidity was defined as the presence of three or more chronic conditions [33].

### Sample size

The study size was defined according to the main Parchemins Study outcome (self-rated health). Considering an effect size of 0.3, a type 1 error of 0.05 and a power of 0.8, the total sample size was set at 352, rounded up to 400 to account for the possible attrition along the study waves as previously described elsewhere [21].

### Statistical analysis

Non-normally distributed continuous variables are presented as median and interquartile range (IQR), whereas categorical variables are listed as proportions with percentages and 95% confidence intervals (95% CI). Continuous variables were compared with the Mann-Whitney test. Categorical variables were compared with the chi-square test or the Fisher’s exact test, as appropriate. The significance level was set at 0.05.

We first conducted bivariate analyses between all variables and the main outcome and retained factors with a *p*-value of 0.2 or lower. We then fitted three regression models whose results are presented as adjusted odds ratio (aOR) with 95% CI. The first model included sociodemographic and migration-related variables. We added the potential confounders related to integration and social support as well as those related to economic resources into a second model. Finally, we introduced variables related to health in the last model.

Missing data concerned 10% of the respondents. We excluded observations from the final analysis in two situations: a) in those with any missing value in the composite outcome; and 2) when more than one value was missing in any of the variables. In the case of a unique value missing in any variable, we replaced it with the mode or median value of the distribution, as appropriate. No variable retained in the analyses presented more than 1.7% of missing values. All analysis were conducted using R (version 3.5.3).

### Ethics approval and consent to participate

All participants provided written informed consent and this study was approved by the Ethics Committee of Geneva Canton, Switzerland (CCER 2017–00897).

### Role of the funding sources

The funding sources had no role in the in study design; in the collection, analysis, and interpretation of data; in the writing of the report; and in the decision to submit the paper for publication. The corresponding author confirms that he had full access to all the data in the study and had final responsibility for the decision to submit for publication.

## Results

### Participants

We included 456 participants, of whom 246 (53.9%) were undocumented. Overall, participants were predominantly women (71.9%) of middle age (43.3; IQR: 15.5 years) from Latin America (63.6%) or Asia (20.2%, mainly The Philippines) who had migrated to Switzerland for economic reason (61.1%) more than 12 (IQR: 7) years prior to the beginning of the study (Table 1). A majority (57.2%) met the criteria for the main outcome of altered mental health and 17.5% suffered multi-morbidity.

**Table 1:** Characteristics of the study participants (*n* = 456) stratified by residence status

	Total (*N* = 456)	Undocumented (*N* = 246)	Regularized (*N* = 210)	*P*-value*
	n (%) or median (IQR)	n (%) or median (IQR)	n (%) or median (IQR)	
Women	328 (71.9%)	175 (71.1%)	153 (72.9%)	0.762
Origin				0.088
Latin America	290 (63.6%)	150 (61.0%)	140 (66.7%)	
Asia	92 (20.2%)	55 (22.4%)	37 (17.6%)	
Non EU/EFTA Europe	39 (8.6%)	17 (6.9%)	22 (10.5%)	
Africa	35 (7.7%)	24 (9.8%)	11 (5.2%)	
Age (years)	43.3 (15.5)	42.2 (14.7)	44.2 (16.7)	0.008
University or tertiary education	104 (22.8%)	59 (24.1%)	45 (21.5%)	0.507
Years in Geneva	12 (7)	10 (7)	13 (5)	< 0.001
Migration mainly for economic purpose	279 (61.1%)	155 (62.8%)	124 (58.9%)	0.122
Visited home country since in Switzerland	238 (52.2%)	108 (43.9%)	130 (61.9%)	< 0.001
Accommodation crowding index	1.50 (1)	2 (1.5)	1.25 (1)	< 0.001
Social network (*n* > 3)	219 (48%)	103 (41.9%)	116 (55.2%)	0.006
Social isolation (very or rather)	131 (28.7%)	95 (38.6%)	36 (17.1%)	< 0.001
Abuse	103 (22.6%)	57 (23.2%)	46 (21.9%)	0.834
Discrimination	147 (32.2%)	93 (37.8%)	54 (25.7%)	0.008
Inability to pay CHF 1500	301 (66.0%)	192 (78.0%)	109 (51.9%)	< 0.001
Remittance to home country	297 (65.1%)	159 (64.8%)	138 (65.9%)	0.789
Working hours per week	32 (24)	25 (29.5)	36.5 (18.8)	< 0.001
Health insurance	137 (30.0%)	32 (13.0%)	105 (50.0%)	< 0.001
Multi-morbidity	80 (17.5%)	52 (21.1%)	28 (13.3%)	0.039
Altered mental health	261 (57.2%)	164 (66.7%)	97 (46.2%)	< 0.001

* *p*-value concerns differences between regularized and undocumented migrants

Regarding the living, social and working conditions, the median number of occupants per bedroom was 1.5. While 48% could rely on at least three persons of trust, 28.7% felt rather or very isolated. Exposure to abuse (22.6%) and discrimination (32.6%) was reported by a minority of participants. Despite an average workload of 32 (SD: 24) weekly working hours, only 34% had sufficient financial reserve to cover an expense of CHF 1500 at short notice. Only 30% had a health insurance.

There were significant differences between groups in disfavor of the undocumented group for most social, economic and health-related variables at the exception of the level of education, exposure to abuse, and sending money back home.

### Prevalence of mental health conditions

Table 2 displays the prevalence of the components of the main outcome. While 18.4% (95% CI: 15.0–22.3%) reported a recent diagnosis of anxiety or depression by a health professional, almost two times more participants screened positive for symptoms evocative of these conditions. Indeed, 36% (95% CI: 31.6–40.6%) and 45.4% (95% CI: 40.8–50.1%) reported symptoms for anxiety and depression, respectively. Most participants presented mild or moderate symptoms for both conditions. In addition, 23% (95% CI: 19.2–27.2) reported sleep disturbance. For all three conditions, undocumented migrants fared significantly worse than regularized participants.

**Table 2:** Prevalence and severity of mental health conditions in the study participants (*n* = 456) stratified by residence status

	Total (*N* = 456)			Undocumented (*N* = 246)			Regularized (*N* = 210)			*P*-value*
	n	%	95% CI	n	%	95% CI	n	%	95% CI	
Known diagnosis of anxiety or depression	84	18.4	15.0–22.3	64	26.0	20.6–32.0	20	9.5	5.9–14.3	< 0.001
Symptoms of depression (PHQ-9)	207	45.4	40.8–50.1	137	55.7	49.2–62.0	70	33.3	27.0–40.1	< 0.001
Mild	122	26.8	22.7–31.1	73	29.7	24.0–34.8	49	23.3	17.8–29.6	
Moderate	60	13.2	10.2–16.6	42	17.1	12.6–22.4	18	8.6	5.2–13.2	
Moderately severe to severe	25	5.5	2.6–8.0	22	8.9	5.7–13.2	3	1.4	0.3–4.1	
Symptoms of anxiety (GAD-7)	164	36.0	31.6–40.6	109	44.3	38.0–50.8	55	26.2	20.4–32.7	< 0.001
Mild	96	21.1	17.4–25.1	54	22.0	16.9–27.7	42	20.0	14.8–26.1	
Moderate	55	12.1	9.2–15.4	43	17.5	12.9–22.8	12	5.7	3.0–9.8	
Severe	13	2.9	1.5–4.8	12	4.9	2.5–8.4	1	0.5	0.0–2.6	
Sleep disturbance (PSQI)	105	23.0	19.2–27.2	79	32.1	26.3–38.3	26	12.4	8.2–17.6	< 0.001
Fairly bad	87	19.1	15.6–23.0	67	27.2	21.8–33.3	20	9.5	5.9–14.3	
Bad	18	3.9	2.4–6.2	12	4.9	2.5–8.4	6	2.9	1.1–6.1	

* *p*-value concerns differences between regularized and undocumented migrants

### Factors associated with altered mental health

In the baseline model of multivariable analysis, the lack of residence permit (aOR: 2.2; 95% CI: 1.4–3.3), Latin American versus Asian (aOR: 0.5; 95%CI: 0.3–0.9) or European (aOR: 0.3; 95% CI: 0.1–0.7) origin and younger age (aOR: 0.7; 95% CI: 0.5–0.9) were significantly associated with altered mental health (Table 3). After adding the socioeconomic variables into the model, the association with residency status and origin lost its significance. At this stage, self-reported social isolation (aOR: 2.7; 95% CI: 1.6–4.7), exposure to abuse (aOR: 1.9; 95% CI: 1.1–3.5) or discrimination (aOR: 1.7; 95% CI: 1.1–2.8) and the lack of financial reserve (aOR: 2.3; 95% CI: 1.4–3.8) showed a significant association with altered mental health. Moreover, living in a crowded accommodation (aOR: 1.3; 95% CI: 1.0–1.6, *p*-value 0.051) tended to increase the risk of altered mental health. In the model including all variables, health-related factors did not significantly alter the relationships previously observed at the exception of the exposure to discrimination (aOR: 1.6; 95% CI: 1.1–2.7) whose association with the main outcome became borderline. Having a health insurance had no effect on mental health but suffering from multi-morbidity (aOR: 3.2; 95% CI: 1.7–6.5) showed strong association with the main outcome.

**Table 3:** Multivariate associations between altered mental health and demographic, migration-related, social, economic and health factors

	Model 1		Model 2		Model 3	
	aOR (95% CI)	*p*-value	aOR (95% CI)	*p*-value	aOR (95% CI)	*p*-value
Lack of residence permit	2.2 (1.4, 3.3)	< 0.001	1.4 (0.9, 2.3)	0.137	1.3 (0.8, 2.2)	0.259
Origin (reference Latin America)						
Asia	0.5 (0.3, 0.9)	0.021	0.6 (0.3, 1.1)	0.120	0.6 (0.3, 1.2)	0.136
Europe	0.3 (0.1, 0.7)	0.006	0.5 (0.2, 1.2)	0.138	0.5 (0.2, 1.3)	0.182
Africa	1.2 (0.5, 2.9)	0.638	0.8 (0.3, 1.9)	0.564	0.9 (0.4, 2.8)	0.852
Men	0.7 (0.4, 1.1)	0.121	0.7 (0.4, 1.2)	0.197	0.8 (0.4, 1.3)	0.332
Age (per additional decade)	0.7 (0.5, 0.9)	0.001	0.7 (0.6, 0.9)	0.015	0.7 (0.5, 0.9)	0.007
Duration in Geneva (per additional year)	1.0 (1.0, 1.1)	0.746	1.0 (1.0, 1.1)	0.267	1.0 (1.0, 1.1)	0.493
Visit to home Country	0.7 (0.5, 1.2)	0.178	0.7 (0.4, 1.1)	0.141	0.7 (0.4, 1.1)	0.127
Crowding Index (per additional unit)			1.3 (1.0, 1.6)	0.051	1.2 (1.0, 1.6)	0.065
Social Network (> 3)			1.0 (0.7, 1.6)	0.973	1.1 (0.7, 1.7)	0.722
Social isolation (very or rather isolated)			2.7 (1.6, 4.7)	< 0.001	2.4 (1.4, 4.2)	0.001
Abuse			1.9 (1.1, 3.5)	0.032	1.9 (1.1, 3.5)	0.034
Discrimination			1.7 (1.1, 2.8)	0.030	1.6 (1.0, 2.7)	0.057
Inability to face unexpected bills			2.3 (1.4, 3.8)	< 0.001	2.2 (1.4, 3.6)	0.001
Hours of work (per additional hour)			1.0 (1.0, 1.0)	0.239	1.0 (1.0, 1.0)	0.257
Health Insurance					1.0 (0.6, 1.7)	0.993
Multi-morbidity					3.2 (1.7, 6.5)	< 0.001
Model fit indices						
Akaike Information Criteria	588.28		543.41		534.58	
McFadden’s pseudo-R2	0.084		0.178		0.199	
Likelihood ratio test			X^2^ (7)=58.768	< 0.001	X^2^ (2)=12.932	0.002

Both the likelihood ratio tests and the McFadden’s pseudo-R2 show a clear improvement of fit from a model to another (Table 3). Of interest, the McFadden’s pseudo-R2 of our third model, which is 0.199, indicates a very good fit.

## Discussion

This study showed that a majority (57%) of long-established economic migrants, undocumented or in the process of obtaining a residence permit, presented symptoms of at least one mental health condition, most frequently anxiety and depression. While undocumented migrants displayed overall higher prevalence of symptoms of mental health conditions compared to migrants in the process of regularization, the absence of residency permit per se was not a significant predictor of altered mental health when adjusting for measures of integration, social support and economic resources at the onset of the new policy implementations. Indeed, younger age, exposure to abuse and discrimination, strong feeling of social isolation, lack of financial reserve and multimorbidity were all associated with altered mental health.

Undocumented migrants in Europe, including youth, rate their quality of life and their health lower than legal residents [9, 17, 34, 35]. Mental health, while often overlooked in studies, seem to play a key role in such self-evaluation [7]. We found that 57.2% of the study participants presented symptoms of at least one mental health condition, while 45.4% screened positive for symptoms of depression and 36% of anxiety. Comparison with previous studies conducted in Europe is hazardous considering their scarcity and differences in migrants’ profiles and research methodology. Only few studies have included participants recruited in the community rather than in health services. Herren et al. found similar prevalence in a small sample of undocumented migrants in Zurich, Switzerland [11]. Schoever et al. showed that female migrants in the Netherland were unlikely to seek healthcare for mental health problems despite high proportion of positive screening for anxiety (78%), depression (64%) and sleeping disorder (71%) [9]. Andersson et al. found prevalence of similar extent in Sweden among undocumented migrants with a frequent asylum background and living in very unstable conditions [12]. Studies conducted in healthcare setting frequently found even higher prevalence [18, 36]. Of concern, we found a significant discrepancy between the proportions of participants presenting altered mental health and of those who had been diagnosed which such conditions prior to the survey. Moreover, 17.5% reported suffering from multiple chronic conditions despite their young age, which confirms previous findings [37]. Our results support that mental health is likely to represent a substantial and often unrecognized share of the burden of ill health affecting this group of population, which needs to be tackled by multi-pronged interventions.

The description of the study population highlights the daily challenges migrants were facing in the context of the new regularization policy. Most participants had been living and working in Geneva for many years but were still presenting insecurity in diverse life domains. Indeed, a large proportion lived in overcrowded housing, showed financial insecurity and lacked access to the healthcare system. Those in the regularization process or recently regularized tended to fare slightly better, reflecting the selection process leading to eligibility to apply for a residency permit. Overall, exposure to abuse, and to discrimination to a lesser extent, social isolation and the inability to pay a CHF 1500.- bill at short notice were all predictors of altered mental health. This confirms previous studies showing the negative impact of cumulative social, professional and economic stress on mental health in undocumented migrants while effective social support and engagement in community activities appeared to be protective [8, 12, 18, 34, 36]. Some authors have suggested that precarious undeclared employment conditions played a central role in the chain of events leading to altered health in this group [4, 15, 16]. Castaneda framed the negative effects of these social and legal factors as the “*illegal syndrome”* in the context of their frequent co-occurrence with mental conditions such as anxiety and depression [38]. Our results tended to confirm these findings considering the high proportion of participants facing cumulated stress. Of interest, migrants in the process of or recently regularized did not present overall higher odds of better mental health. This is possibly due to the still too short duration of exposure to the potential advantages of the residence status for protective effects on life insecurities to unfold. In other settings, integration policies have shown a positive effect on migrants’ health and wellbeing over the medium to longer term [39]. The next Parchemins study data collection waves will allow for better understanding of the mid-term impact of obtaining a residence permit and thus of the access to social protection on mental health. Of note, while suffering multiple chronic health conditions was strongly associated with altered mental health, better access to the healthcare system through having a health insurance showed no association with the mental health status. This may indicate that gaining easier access to the healthcare system through the regularization may not be sufficient to offset the health disadvantages and that interventions on non-health factors may prime.

Several limitations must be taken into account when interpreting the results of this study. First, our study participants are not fully comparable with other groups of undocumented migrants such as those recently arrived in Europe who had not enough time to set up a relatively stable situation. Indeed, a significant proportion of undocumented migrants is highly mobile and face great difficulty in accessing to even basic employment and housing [40]. In that sense, future studies targeting recently settled undocumented immigrants may be necessary to complement the understanding of mental health issues in this group. Second, undocumented migrants are not a homogenous population across Europe and national immigration policies define who becomes undocumented and how. For instance, undocumented groups in Geneva only include few rejected asylum seekers unlike in other cities. Therefore, our results may not be fully comparable with the situation found in other European cities. Developing a comparative study in different cities and regions may prove useful to better apprehend the global burende of mental health. On the other hand, the diversity of our sample gives strength to our results. Third, we used a non-probabilistic sampling which affects the representativeness of our study population. Yet, we set up various strategies to reach a sample as diverse as possible. Fourth, we may have overestimated prevalence of mental health conditions by using cut-off scores of 5 with the GAD-7 and PHQ-7 scales. In clinical setting, cut-off of 10 may be used to diagnose general anxiety or major depression. Yet, we based our analysis plan on similar cut-offs previously used by other researchers in the general population or in primary care setting [25, 41, 42]. Finally, measuring mental health at a different time points is likely to incur fluctuating results considering the impact of macro political, social and economic environment. For instance, the 2020 Covid-19 crisis is likely to negatively impact migrants’ mental health because of its societal consequences. In that sense, regular monitoring of mental health in this population is necessary to identify emerging needs and the role of contextual factors such as the COVID-19 pandemic. In that sense, prospective studies using mixed-methods such as Parchemins may bring valuable insight.

## Conclusions

This is one of the largest study ever conducted in Europe among this group of migrants. It highlights the need to implement social and health policies and programs fostering better mental health in this highly vulnerable group of population by acting on different determinants of their living conditions and general health. On the short term, regularization per se is not sufficient to improve mental health in absence of improvement in other domains of life. Embedded into regularization programs, complementary policies should aim to tackle unfair working conditions and salaries by better employers’ information and closer surveillance of the informal job sectors, to ensure access to adequate housing, to strengthen social integration by reducing the fear to participate in public activities and to provide protection against abuse and discrimination. Moreover, ensuring timely and effective access to health interventions to prevent the early occurrence of chronic conditions is a priority. Providing more and better social and economic securities through legal status regularization may represent a key step towards better health and wellbeing. More research is needed to assess the long-term impact of legal status regularization on mental health.

## Data Availability

The full dataset fully will be made publicly available one year after the Parchemins Study termination (2023) in accordance with the Swiss National Foundation for Scientific Research policy. Data will be available from the corresponding author at yves.jackson@hcuge.ch. The study protocol has been published elsewhere and is freely available at doi:10.1136/bmjopen-2018-028336.

## Abbreviations

95% CI: 95% confidence interval
aOR: adjusted Odd Ratio
CHF: Swiss Francs;
GAD-7: 7-Item general anxiety disorder scale
HUG: Geneva University Hospital
IQR: Interquartile range
NGO: Non-governmental organization
PHQ-9: 9-item Patients health questionnaire
PSQI: Pittsburgh Sleep Quality Index
PTSD: Post-traumatic stress disorder
